# Supplementary figures and images for: miR-194-3 Regulates Proliferation and Apoptosis of Follicular Granulosa Cells by Targeting CHD4 in Zhedong White Geese
Source: Animals (Basel). 2025 Sep 12;15(18):2676. doi: 10.3390/ani15182676 (PMC12466677; doi:10.3390/ani15182676)

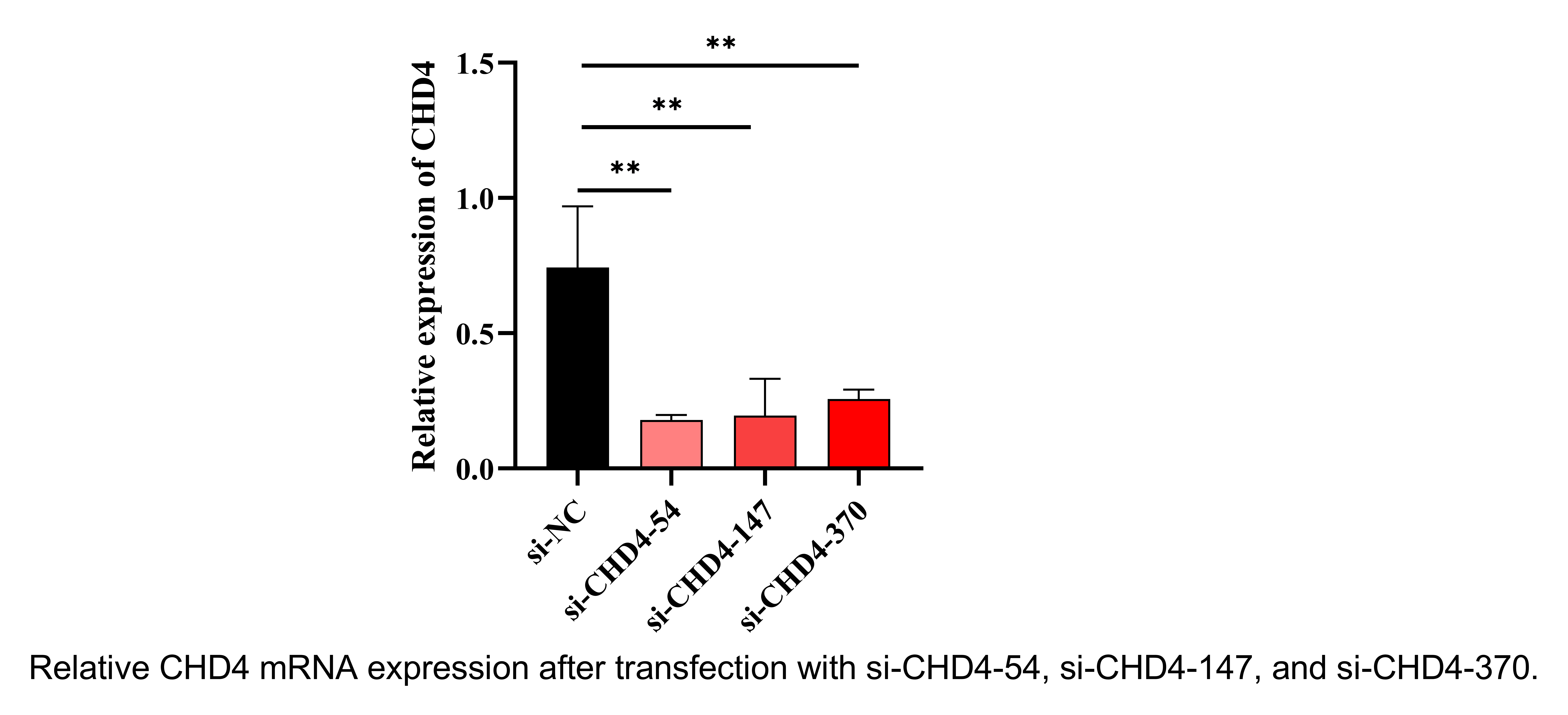

Supplement: Supplementary file 1 [file animals-15-02676-s001.zip › Figure S1.tif]

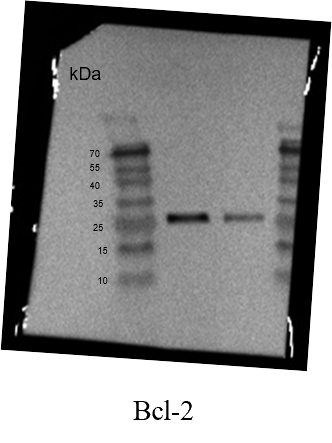

Supplement: Supplementary file 1 [file animals-15-02676-s001.zip › inhibitor Bcl-2.tif]

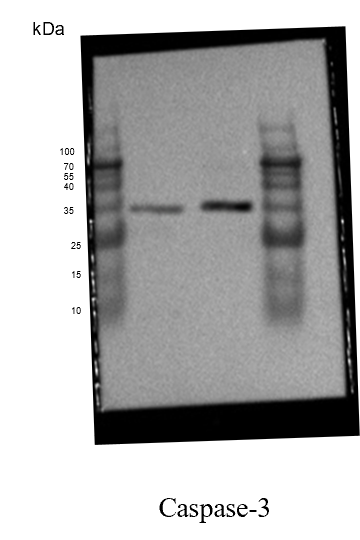

Supplement: Supplementary file 1 [file animals-15-02676-s001.zip › inhibitor Caspase-3.tif]

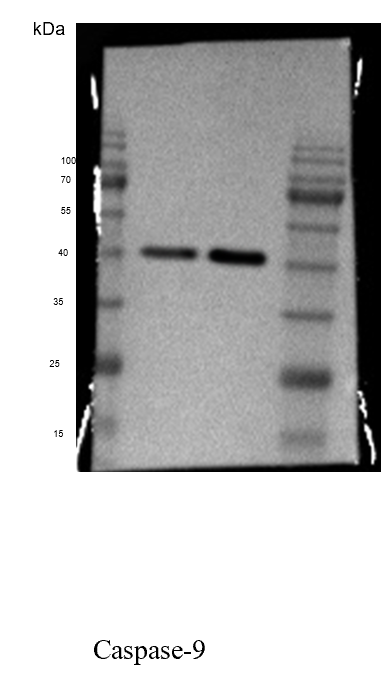

Supplement: Supplementary file 1 [file animals-15-02676-s001.zip › inhibitor Caspase-9.tif]

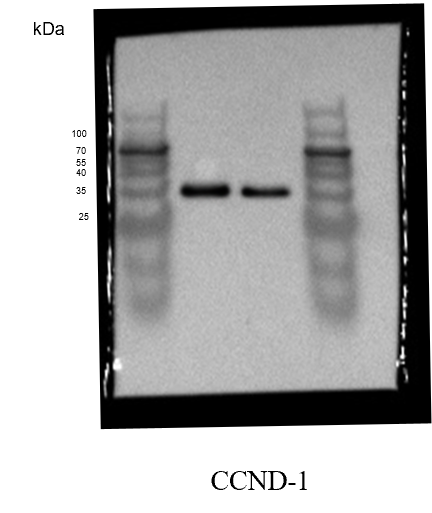

Supplement: Supplementary file 1 [file animals-15-02676-s001.zip › inhibitor CCND-1.tif]

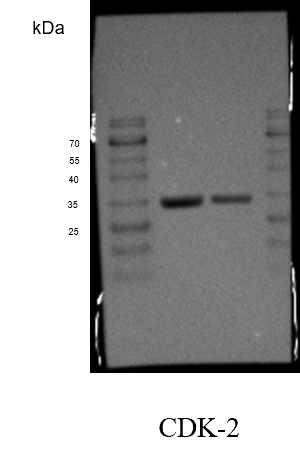

Supplement: Supplementary file 1 [file animals-15-02676-s001.zip › inhibitor CDK-2.tif]

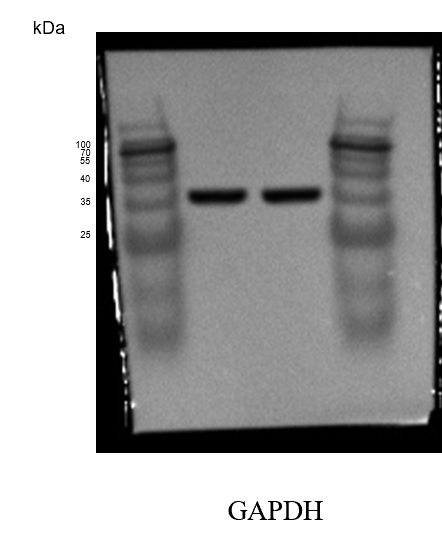

Supplement: Supplementary file 1 [file animals-15-02676-s001.zip › inhibitor GAPDH.tif]

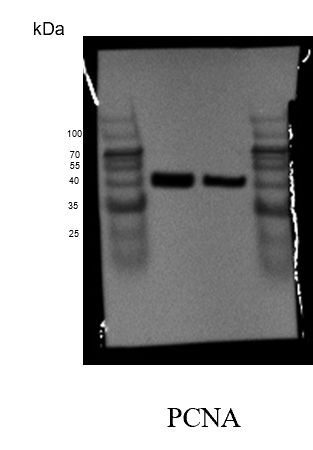

Supplement: Supplementary file 1 [file animals-15-02676-s001.zip › inhibitor PCNA.tif]

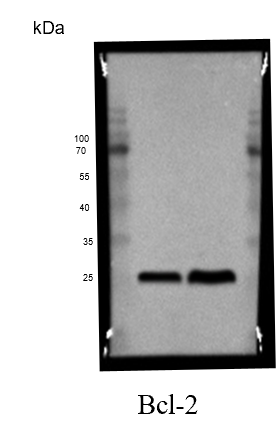

Supplement: Supplementary file 1 [file animals-15-02676-s001.zip › mimic Bcl-2.tif]

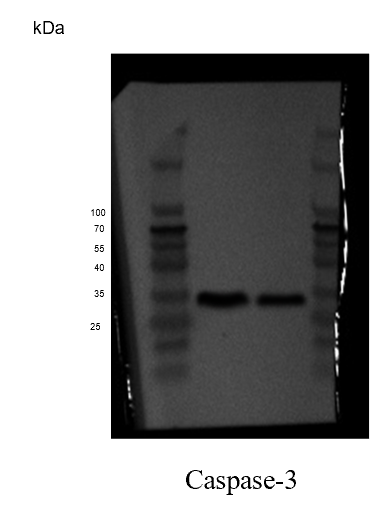

Supplement: Supplementary file 1 [file animals-15-02676-s001.zip › mimic Caspase-3.tif]

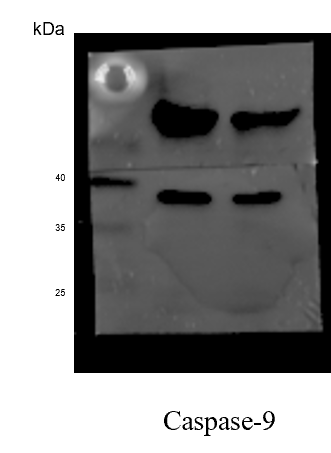

Supplement: Supplementary file 1 [file animals-15-02676-s001.zip › mimic Caspase-9.tif]

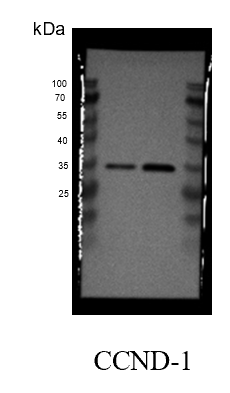

Supplement: Supplementary file 1 [file animals-15-02676-s001.zip › mimic CCND-1.tif]

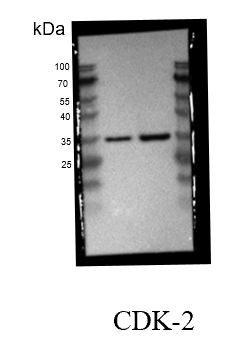

Supplement: Supplementary file 1 [file animals-15-02676-s001.zip › mimic CDK-2.tif]

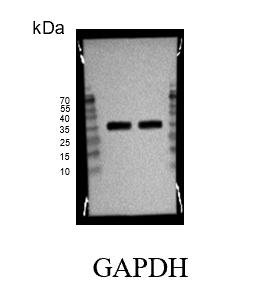

Supplement: Supplementary file 1 [file animals-15-02676-s001.zip › mimic GAPDH.tif]

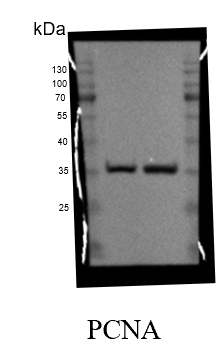

Supplement: Supplementary file 1 [file animals-15-02676-s001.zip › mimic PCNA.tif]

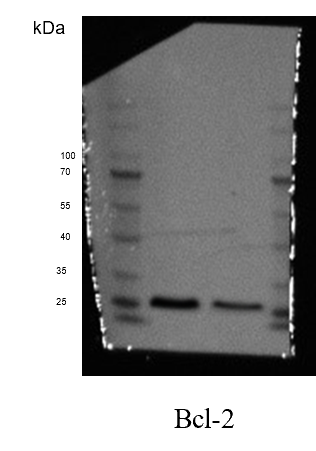

Supplement: Supplementary file 1 [file animals-15-02676-s001.zip › si-CHD4 Bcl-2.tif]

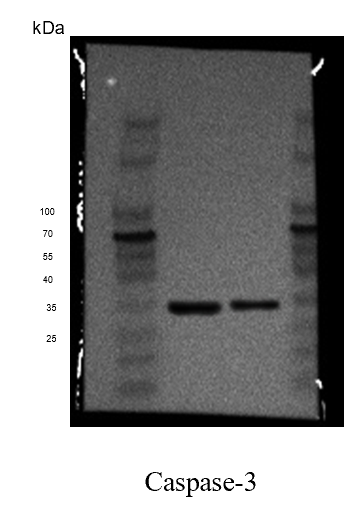

Supplement: Supplementary file 1 [file animals-15-02676-s001.zip › si-CHD4 Caspase-3.tif]

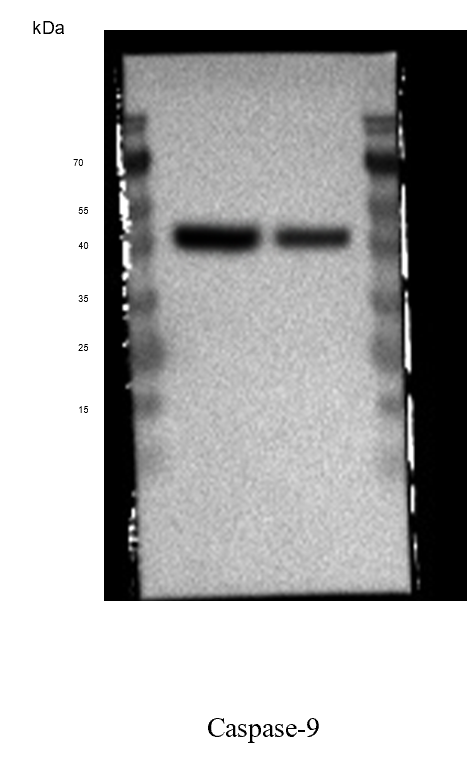

Supplement: Supplementary file 1 [file animals-15-02676-s001.zip › si-CHD4 Caspase-9.tif]

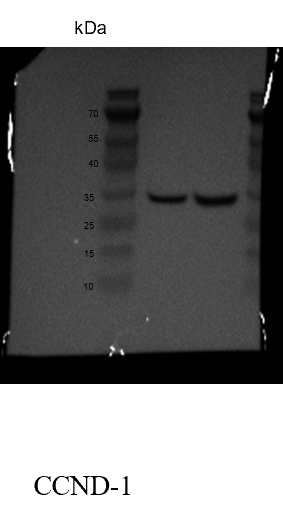

Supplement: Supplementary file 1 [file animals-15-02676-s001.zip › si-CHD4 CCND-1.tif]

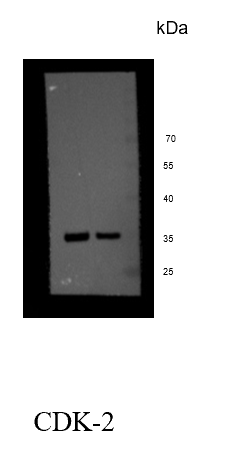

Supplement: Supplementary file 1 [file animals-15-02676-s001.zip › si-CHD4 CDK-2.tif]

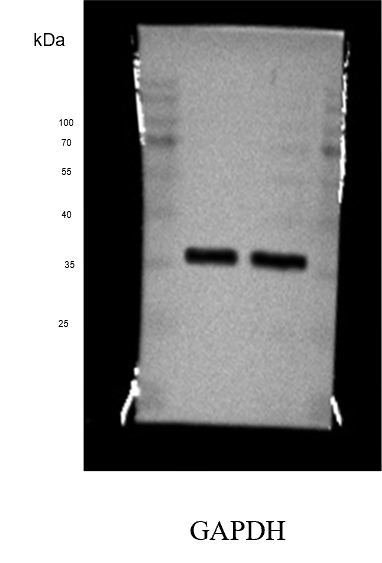

Supplement: Supplementary file 1 [file animals-15-02676-s001.zip › si-CHD4 GAPDH.tif]

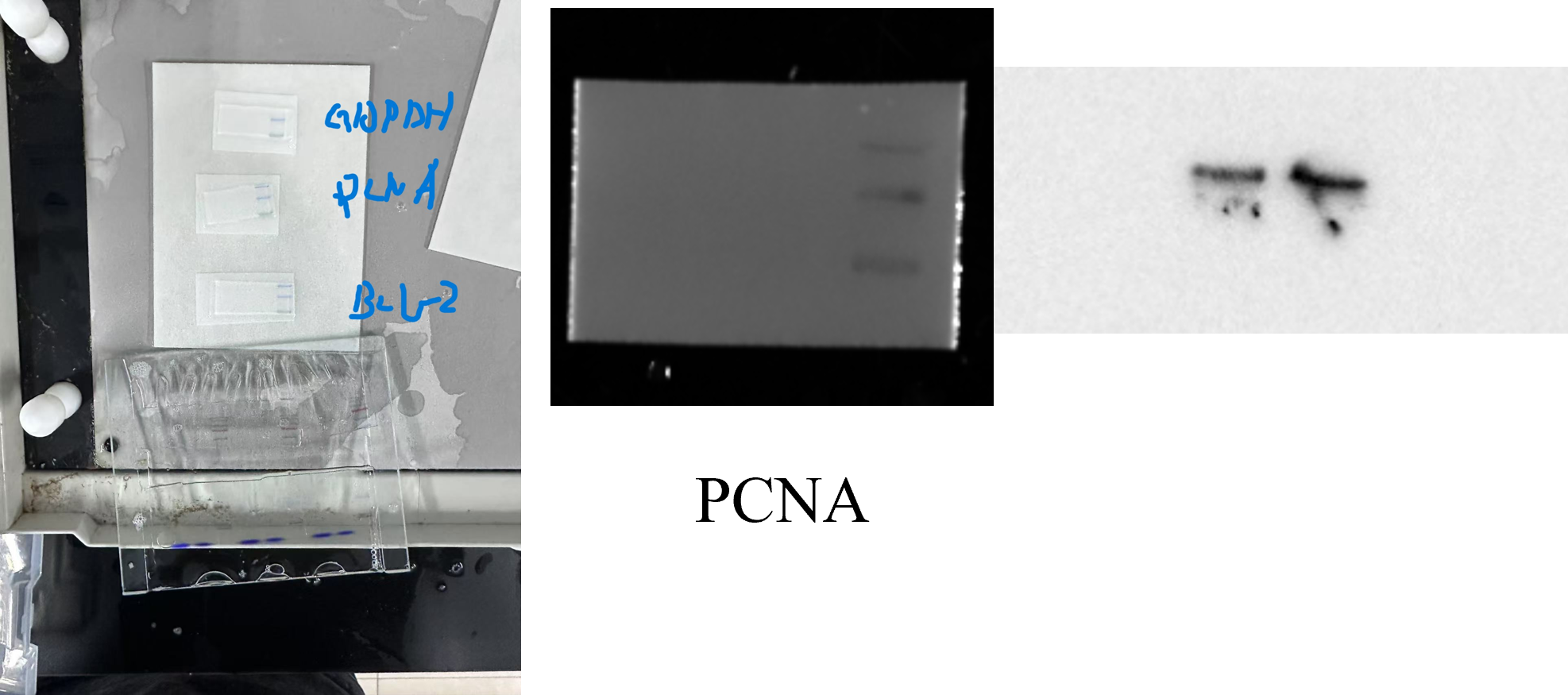

Supplement: Supplementary file 1 [file animals-15-02676-s001.zip › si-CHD4 PCNA.png]
